# Independent evaluation of an in‐house brachytherapy treatment planning system using simulation, measurement and calculation methods

**DOI:** 10.1120/jacmp.v13i2.3687

**Published:** 2012-03-08

**Authors:** M. A. Mosleh Shirazi, R. Faghihi, Z. Siavashpour, H.A. Nedaie, S. Mehdizadeh, S. Sina

**Affiliations:** ^1^ Center for Research in Medical Physics and Biomedical Engineering Shiraz University of Medical Sciences Shiraz; ^2^ Department of Radiotherapy and Oncology Shiraz University of Medical Sciences Shiraz; ^3^ Medical Radiation Department, School of Mechanical Engineering Shiraz University Shiraz; ^4^ Radiation Research Center, School of Mechanical Engineering Shiraz University Shiraz Iran; ^5^ Department of Radiotherapy, Cancer Institute Tehran University of Medical Sciences Tehran Iran

**Keywords:** brachytherapy, treatment planning, Selectron, MCNP, PLATO

## Abstract

Accuracy of treatment planning systems may significantly influence the efficacy of brachytherapy. The purpose of this work is a detailed, varied and independent evaluation of an in‐house brachytherapy treatment planning software called STPS. Operational accuracy of STPS was investigated. Geometric tests were performed to validate entry and reconstruction of positional information from scanned orthogonal films. MCNP4C Monte Carlo code and TLDs were used for simulation and experimental measurement, respectively. STPS data were also compared with those from a commercial planning system (Nucletron PLATO). Discrepancy values between MCNP and STPS data and also those of PLATO and STPS at Manchester system dose prescription points (AL and AR) of tandem and ovoid configurations were 2.5%±0.5% and 5.4%±0.4%, respectively. Similar results were achieved for other investigated configurations. Observed discrepancies between MCNP and STPS at the dose prescription point and at 1 cm from the tip of the vaginal applicator were 4.5% and 25.6% respectively, while the discrepancy between the STPS and PLATO data at those points was 2.3%. The software showed submillimeter accuracy in its geometrical reconstructions. In terms of calculation accuracy, similar to PLATO, as attenuation of the sources and applicator body is not considered, dose was overestimated at the tip of the applicator, but based on the available criteria, dose accuracy at most points were acceptable. Our results confirm STPS's geometrical and operational reliability, and show that its dose computation accuracy is comparable to an established commercial TPS using the same algorithm.

PACS number: 87.53.Jw

## I. INTRODUCTION

Treatment planning has now become an integral part of brachytherapy. The main purpose of treatment planning is to obtain an optimized dose distribution. Therefore, accuracy of the treatment planning software may have a significant effect on the efficacy of the treatment. The Selectron remote afterloading low dose rate (LDR) unit (Nucletron Trading BV, Veenendaal, The Netherlands) coupled with the vaginal cylindrical applicators and the Fletcher‐Suit‐Delclos (FSD) tandem and ovoids are still widely used for most vaginal and cervical cancer treatments in some countries and research work on this is still ongoing.^(^ ^1^ ^–^ ^7^ ^)^

The dose rate distributions around the above‐mentioned applicators loaded with specific configurations of active and inactive pellets are usually calculated by treatment planning systems (TPSs), whereby each pellet is considered as a point source and the dose contributions from the active pellets are summed to determine the dose distribution around the applicator.

The Selectron treatment planning software (STPS) is an in‐house TPS. It determines the dose distribution around different combinations of sources and spacers by assuming each active pellet as a point source. The Nucletron PLATO treatment planning system (Version UPS: 11.3) is also a treatment planning system that uses the same dose calculation formalism.^(8)^

Such treatment planning systems, which are based on simple superposition, ignore the intersource attenuation and scattering of photons in the applicator. Different investigators have studied the shielding effects of applicators and inactive pellets on dose distribution around different applicators with various designs inside the applicators both experimentally and using Monte Carlo (MC) calculations. MC techniques have been widely used as a powerful tool for brachytherapy dosimetry to complement experimental measurements, for example, in characterization of the perturbing effects of applicators.^(^ ^4^ ^,^ ^5^ ^,^ ^9^ ^–^ ^12^ ^)^

The purpose of this study is independent evaluation of the STPS brachytherapy treatment planning software for the first time, by comparing its results with those of PLATO, MC simulations, and thermoluminescence dosimetry (TLD). The authors of this paper have performed this investigation as users of the software and have had no involvement with its development carried out at a different center. It should be noted that no previous scientific paper exists on the STPS software. The performance of the STPS software was evaluated by comparing the dose distribution around different combinations of sources with those calculated by PLATO and the MCNP4C MC code. Finally, TLD measurements were also performed.

## II. MATERIALS AND METHODS

### A.1 Source geometry and applicators

The Nucletron Selectron low‐dose‐rate remote afterloading intracavitary system delivers the prescribed dose via a preselected sequence of active and dummy (inactive) pellets loaded into stainless steel applicator sets through a plastic catheter. The active pellets are composed of 1.5 mm diameter spherical active core covered by 0.5 mm stainless steel.^(^ ^8^ ^,^ ^13^ ^,^ ^14^ ^)^ The Selectron applicator sets (Fletcher‐Suit‐Delclos) were used in this study.

Six patients treated with typical pellet train configurations in tandem, ovoid, and vaginal cylinder applicators were included in this study: three cylinders $(C_{1},\;C_{2}$, and $C_{3})$, two tandem‐ovoid sets $(T_{1}$ and $T_{2})$, and one ovoid $\left(O_{1}\right)$. The dose distributions around each configuration were obtained using the two treatment planning systems (PLATO and STPS) and MC calculations. Experimental measurements using TLDs were also performed for a typical combination of pellets within a vaginal applicator planned for one of the patients.

### A.2 STPS and PLATO

The STPS system uses a point source approximation for calculations regarding spherical sources, and the dose distribution around the source in a homogeneous water phantom is estimated as follows:

(1) $$\dot{D}=\frac{\Gamma \times A\times S(d)\times f_{\lambda }}{d^{2}}$$

Where Γ is the exposure rate constant $\left(R.{\text{cm}}^{2}.{\text{mCi}}^{-1}.{\text{hr}}^{-1}\right)$, *A* is the source activity, $f_{\gamma }$ is the roentgen‐to‐rad conversion factor, *d* is the point to source distance, and *S(d)* is the correction factor for absorption and scatter effects of photons in water. The absorption and scattering correction factors suggested by Meisberger and Van Kleffen and Star are used for S(d).^(15)^ The self‐absorption of the source and the applicator photon absorption are ignored.

**Figure 1:**
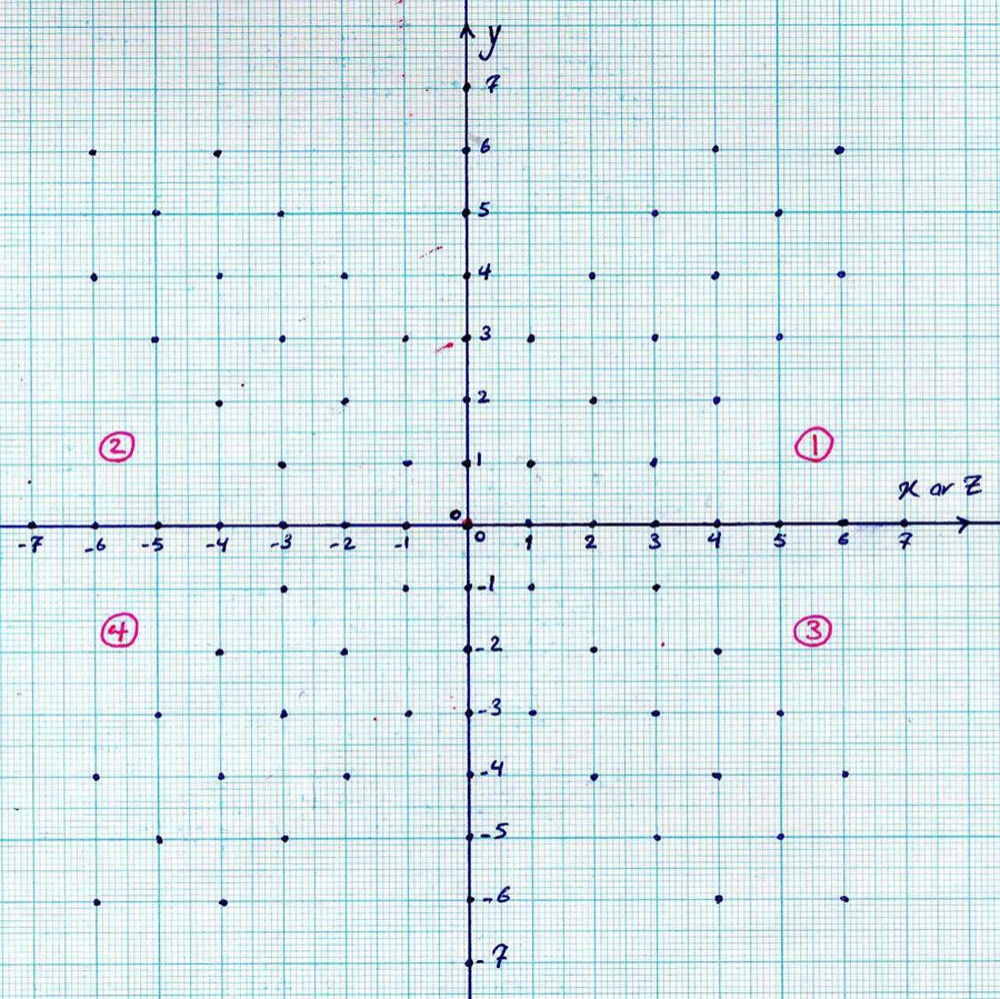
The source point pattern used for determination of the geometrical accuracy of the STPS.

Scanned anterioposterior (AP) and lateral radiographs of a patient with the applicator inside their body are given to the software as input, and then the positions of the active pellets are identified by clicking on each source on both images within STPS. The geometrical accuracy of the software in determining the active source positions was checked using a graph paper. To do this, 56 possible 2D positions of the active sources on each of the XY and YZ planes were marked on a graph paper (Fig. 1). The graph paper was scanned and used as the input of the STPS software. After determination of the center point (origin of coordinate system) on the graph paper, the positions of points with known coordinates were determined using the STPS localization module. The percentage differences between the real positions and the positions identified by STPS were used for checking the geometrical accuracy of the software.

Using the superposition method, the software calculates the dose rate distribution around a configuration of sources. The dose rate calculation formalism of Nucletron PLATO (Version UPS: 11.3) treatment planning system (TPS) and that of STPS are very similar.

Independent calculation of dose at a distance away from the source(s) in tissue using first principles is fundamental to brachytherapy TPS commissioning. To evaluate the dose calculation algorithm used in the STPS software, a program was written using the MATLAB software (version 7.2) (The MathWorks, Natick, MA), based on the above‐mentioned dose calculation algorithm. The output results of this program were then compared with those of STPS.

### A.3 Monte Carlo calculations

The Monte Carlo N‐particle transport code MCNP4C^(16)^ was used to calculate the dose distribution around each configuration of sources for the six patients. To consider the full scattering condition, a spherical water phantom with dimension of 40 cm, containing full geometry of the applicator, spacers, and sources, was simulated. The *F8 tally, which computes the amount of energy deposited inside the tally cells, was used to score the absorbed energy (MeV) inside the spherical tally cells of dimension less than 0.15 cm. Each program was run for 10^8^ starting particles to obtain a standard deviation of less than 3% in the tallied quantity.

**Figure 2:**
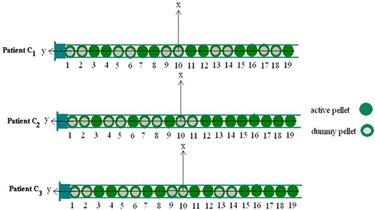
The source train used in treatment of three patients.

The simulation geometry of source configurations inside the vaginal applicator used in treatment of the three patients $(C_{1},\;C_{2}$ and $C_{3})$ is shown in Fig. 2.

**Figure 3:**
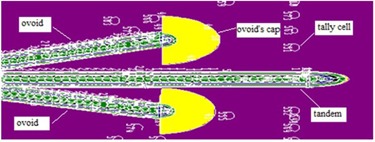
The XY plane of the simulated geometry used in treatment of a patient using a tandem‐ovoid applicator set $\left(T_{1}\right)$.

Two of the six patients $(T_{1}$ and $T_{2})$ were treated with a tandem‐ovoid applicator set, each with a unique source configuration, and the last patient $\left(O_{1}\right)$ was treated using only ovoids. All geometry components such as source trains and applicators used for treatment of the patients were simulated in this study with exact details obtained from two scanned orthogonal radiographic film (AP and lateral). The origin of the simulation coordinates was considered at the cervical os, in the same way as in normal routine planning. The dose rate at spherical tally cells located at different distances were obtained using the *F8 tally. The simulation geometry of one of the treatments using a tandem‐ovoid set is shown in Fig. 3.

### A.4 Experimental measurements

**Figure 4:**
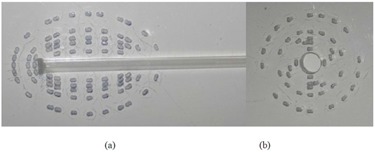
The central slab of the two phantoms to measure in the: a) XY plane, and b) YZ plane.

**Figure 5:**
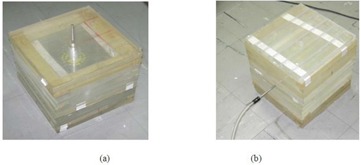
The phantom configurations at the time of irradiations to measure in the: a) XY plane, and b) YZ plane.

Experimental measurements were performed using TLDs for one of the considered treatment configurations using a vaginal (cylinder) applicator (C1). For this purpose, two $30\times 30\times 30\;{\text{cm}}^{3}$ phantoms were constructed from Plexiglas slabs to compare measured dose distributions in both transverse and longitudinal planes with the values obtained by STPS and PLATO for these planes. Several cubic holes with approximate dimensions of $3\times 4\times 1\;{\text{mm}}^{3}$ were drilled on the central slab of the phantom at different distances from the applicator to accommodate the $0.9\times 0.9\times 3\;{\text{mm}}^{3}$ TLD chips (Fig. 4). A cylindrical hole was also machined in the two phantoms for inserting the applicator. When the applicator and the TLD chips were put in place, the applicator was loaded with the source train used for treatment of the first patient, as shown in Fig. 5. Referring to Fig. 2, the most important points for consideration were the prescription ones (A :(1.5, 0, 0) and B :(−1.5, 0, 0)) and those at 1 cm from the applicator tip :(0, 3.8, 0) (because of the high attenuator material in front of photon's path).

It should be added that TLD calibration was done with Cs‐137 teletherapy source of Shiraz University Radiation Research Center. Measurements were chosen to be in the dose ranges of 10–100 cGy for being in the linear ranges of TLD response curve but relative dosimetry was performed in the end.

As mentioned before, both treatment planning systems (STPS and PLATO) calculate dose distribution in a homogeneous water phantom, but the utilized phantom material for experimental measurement was Plexiglas. Therefore, in order to minimize the impact of these two materials on the final results and be able to directly compare all data, MC simulations were done for both phantoms separately and a Plexiglas‐to‐water conversion factor was achieved.

**Figure 6:**
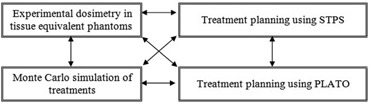
Summary of the intercomparisons in this investigation.

Finally, the data obtained from each of the above‐mentioned methods were compared to evaluate the performance of the STPS software. Figure 6 shows a block diagram which summarizes the intercomparisons performed between the different components in this study.

## III. RESULTS

The STPS software accuracy in determination of source coordinates was evaluated and it was shown that this software has an acceptable operation from the geometrical reconstruction viewpoint. The percentage differences between the real positions in Fig. 1 and the positions determined by the STPS software were found to be less than 1% at all positions.

### A.1 Patients treated with a cylindrical applicator $(C_{1},\;C_{2}$, and $C_{3}$)

**Figure 7:**
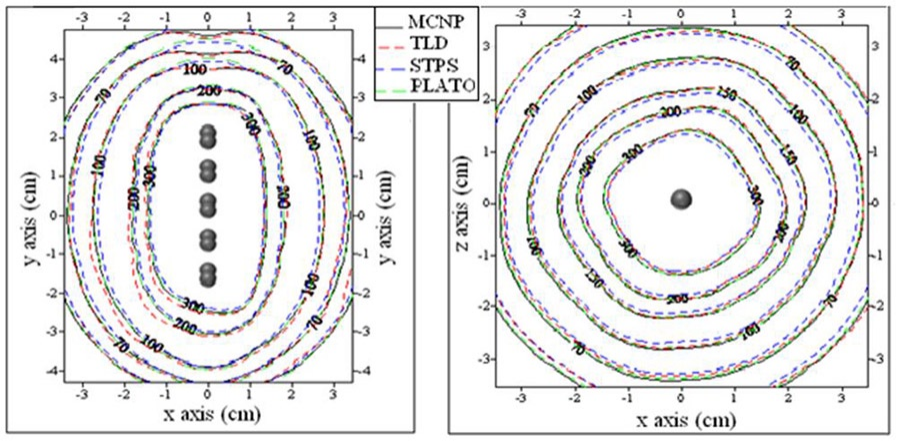
Interpolated isodose contour maps around a cylinder loaded with 10 active sources obtained by MC, STPS, PLATO, and TLD: a) XY plane, and b) YZ plane. (Dose rates values are in units of cGy/hr.)

The dose distribution around three different combinations of the spherical sources inside the cylindrical applicator used for treatment of patients was calculated by MC, STPS, and PLATO calculations. The experimental measurements were also performed for one of the patients using TLD. Figure 7 compares the isodose contour maps in the longitudinal (XY) and transverse (YZ) planes estimated by these three methods. As is obvious from the isodose curves, the point source approximation algorithm (STPS and PLATO) overestimates the dose at the applicator tip. It should be noted that the spheres shown in these figures relate to the approximate positions of active sources in order to give a schematic view, so they are not the exact 3D positions.

Percentage differences between the doses estimated by the four mentioned methods at prescription points A, B, and at a distance of 1 cm from the tip of the applicator are shown in Table 1.

**Table 1:** Percentage differences between the doses obtained by all methods at three points.

$E_{\text{TLD}\&\text{PL}.}$ *(%)*	$E_{\text{ST}.\&\text{PL}.}$ *(%)*	$E_{\text{MC}\&\text{ST}.}$ *(%)*	$E_{\text{TLD}\&\text{ST}.}$ *(%)*	$E_{\text{MC}\&\text{TLD}}$ *(%)*	*Point*
5.7	2.3	4.2	8.1	5.5	A
7.1	2.3	4.8	9.3	3.5	B
33.4	2.3	25.6	30.1	3.6	1 cm from applicator tip

Note: $E_{\left(x\&y\right)}\;(\%)=|(E_{x}-E_{y})/E_{x}|?\;100$

### A.2 Patients treated with tandem‐ovoid applicator $(T_{1}$ and $T_{2})$

**Figure 8:**
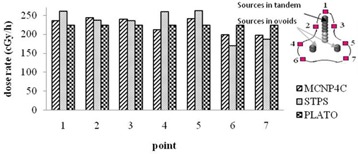
Comparison of the dose rates at different points on the 100% isodose curve within the XY plane for patient $T_{1}$.

The dose distribution around two typical source configurations inside tandem and ovoid applicators $(T_{1}$ and $T_{2})$ were obtained using STPS, PLATO, and MC calculations. Figure 8 shows the values of dose rate calculated by MCNP4C code and STPS at seven points on the 100% isodose curve of PLATO for patient $T_{1}$. The difference of the values of dose rate at the left and right of point A prescription points (AL and AR) between MCNP–STPS, MCNP–PLATO, and PLATO–STPS were 2.5±0.5%,7.4±0.8%, and 5.4±0.4%, respectively. It should be noted that point 1 in Fig. 8 is not located at the tip of the applicator; it is actually located within the XY plane in front of the curvature of the applicator. However, the point at the tip of the applicator is located in the YZ plane and the difference of the dose rates at this point between MCNP–STPS and MCNP–PLATO was found to be 19% and 17%, respectively.

**Figure 9:**
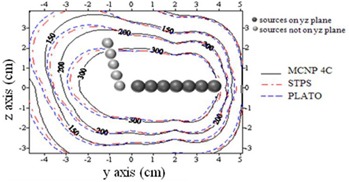
Interpolated isodose contour maps around pellet configurations of patient $T_{2}$ in YZ plane obtained by STPS, PLATO, and MC calculations. Superior (cranial) and posterior directions are toward the right and top of the diagram, respectively. (Dose rates values are in units of cGy/hr).

Isodose contour maps around the sources in the tandem‐ovoid applicator set for patient $T_{2}$ is shown in Fig. 9. The differences between the results of PLATO–MCNP4C and STPS– PLATO at prescription points (AL and AR) were 8.6±0.6% and 8.6±1.0%, respectively.

**Figure 10:**
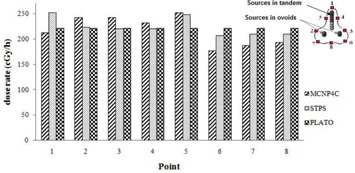
Comparison of the dose rates at different points on the 100% isodose curve in the XY plane for patient $T_{2}$.

Figure 10 shows the values of dose rate calculated by MCNP4C code and STPS at seven points on the 100% isodose curve of PLATO for patient $T_{2}$. As can be seen from the figure, the differences in calculated dose rates at points near the ovoids and at the tip of the applicator are greater than other points. Point 1 is located at the tip of the applicator for patient $T_{2}$, and the difference between MCNP–STPS and MCNP–PLATO results were 16% and 26%, respectively.

### A.3 Patient treated with ovoids (O1)

**Figure 11:**
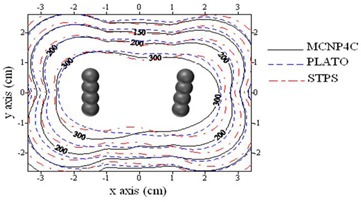
Interpolated isodose contour maps around pellet configurations of patient $O_{1}$ in the XY plane obtained from STPS, PLATO, and MC calculations. (Dose rates values are in units of cGy/hr.)

**Figure 12:**
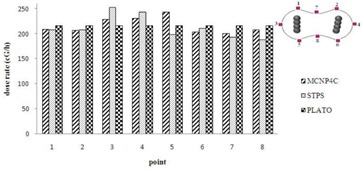
Comparison of the dose rates at different points on the 100% isodose curve in the XY plane for patient $O_{1}$. (Note: each point's coordinate is 1:(−1,1.65,0), 2:(1,1.55,0), 3:(−2.7,0,0), 4:(2.7,0,0), 5:(−1,−1.65,0), 6:(−1,−1.7,0), 7:(0,1.6,0) and 8:(−0,−1.7,0).)

All the above procedures were repeated for the last patient treated using only ovoids. The isodose curves in the XY plane and the dose rates around several points around the sources are shown in Figs. 11 and 12, respectively.

As expected, the maximum difference between the results of PLATO, STPS, and MCNP4C code was observed in planes containing the ovoids. The percentage differences between the results of MC calculations with PLATO and STPS were 4.0%±2% and 6.3%±1.5%, respectively.

## IV. DISCUSSION & CONCLUSIONS

MC techniques have previously been used to study the dosimetry of gynechological brachytherapy. For example, Markman et al.^(14)^ investigated the attenuating effects of a typical intrauterine and double ovoid combination set using an in‐house MC photon transport code for both LDR and HDR brachytherapy. Pérez‐Calatayud et al.^(17)^ used GEANT4 MC code for simulation of a sequence of active and inactive pellets inside a tandem applicator and reported the angular dependency of dose reduction. Sina et al.^(4)^ determined dosimetry parameters of a single spherical source inside the vaginal applicators according to AAPM TG‐43 dose calculation formalism by both TLD measurements and MCNP4C code. They reported an anisotropy function of approximately 0.9 at points located the tip of the applicator.

The discrepancy between the dose estimated by STPS software and the program written in MATLAB for a single source was found to be less than 1% at different distances, which was in accordance with the IAEA‐TRS 430 report acceptability criteria (less than 5%).

The results obtained for the patient treated with cylindrical applicator showed that not taking the intersource, spacers, and applicator effects into consideration would cause significant errors in dose estimation at points located around the tip of the applicator. By simulation of a cylinder applicator, this overestimation was found to be about 30%, which is comparable with the values of 20% and 33% obtained by Fragoso et al.^(5)^ and Gifford et al.^(2)^ using EGSnrc(V2) and MCNPX 2.4k MC codes, respectively. By comparing the results of PLATO and STPS, which do not consider such shielding effects, with the results of MCNP4C simulations and TLD measurements, a good estimate of the attenuation effects is obtained. The good agreement observed between the calculated and measured dose distributions in the transverse (YZ) plane, is mainly due to the low thickness of the shielding material traversed by the photons emitted from the source in these directions. However, the dose distribution calculated by neglecting such effects in the longitudinal (XY) plane, overestimate the dose beyond the tip of the applicator as the photons pass through multiple pellets and thicker shielding.

Note that the impact of phantom material on TLD measurements was considered by MC simulations and Plexiglas‐to‐water conversion factors for each point of study (TLD positions in the phantom) were achieved in the range of 0.9–1.0.

According to the AAPM Task Group #56 (TG 56), for most common LDR sources, the physical dose delivery accuracy of 5%–10% is achievable at distances of 1–5 cm, and also relative to the input data supplied and the algorithm assumed, the computer assisted dose calculations should have a numerical accuracy of at least ±2%. Based on these criteria, it can be concluded that the results obtained by the STPS software are reliable at most of the points around the applicators, except those at the tip of different kinds of applicators (which is the same situation as with the studied version of PLATO). Because of the fact that the areas lateral to the applicator are of the most clinical importance and that the inaccuracy of the STPS software in dose calculation at the applicator's tip has little impact on the global dose prescription, this effect may be considered negligible. Therefore, the software can be used with adequate accuracy at most points.^(18)^

It should be emphasized that neither the authors nor the original developers of STPS had access to the code in PLATO or any previous or subsequent versions of it. The STPS software had been developed based on available formalisms and data available in published scientific literature. The PLATO TPS was used simply as a means of carrying out a comparison. Therefore, evaluating STPS using PLATO constitutes an independent test.

In this paper, we present a detailed and varied set of validation tests of a brachytherapy TPS. In particular, the presented results from TLD measurements and Monte Carlo simulations provide further evidence of the strengths and shortcomings of this type of analytical calculation algorithm utilized in some brachytherapy TPSs. Further, this paper reports on a brachytherapy TPS developed by university‐based researchers that has similar functionality and computational accuracy as an established commercial one.

Work is underway in our research group to produce a CT‐based 3D brachytherapy TPS that incorporates a variety of dose calculation engines (including MC simulation) to suit different situations. The work presented in this paper paves the way for inclusion of the described type of analytical algorithm in that TPS as a simple and fast option.

Although dose calculation accuracy and image import have been improved in more modern systems, our results confirm STPS's geometrical and operational reliability, and show that its dose computation accuracy is comparable to an established commercial TPS using the same algorithm.

## ACKNOWLEDGMENTS

The authors declare no conflict of interest and no financial involvement regarding STPS.

## References

[1] Lakshminarayanan T , Subbaiah KV , Thayalan K , Kannan SE . Suitability of point kernel dose calculation techniques in brachytherapy treatment planning. Med Phys. 2010; 35 (2): 88–99. 10.4103/0971-6203.62202 PMC2884310 20589118

[2] Gifford KA , Mourtada F , Cho SH , Lawyer A , Horton JL, Jr . Monte Carlo calculations of the dose distribution around a commercial gynecologic tandem applicator. Radiother Oncol. 2005; 77 (2): 210–15. 16216363 10.1016/j.radonc.2005.09.006

[3] Parsai EI , Zhang Z , Feldmeier JJ . A quantitative three‐dimensional dose attenuation analysis around Fletcher‐Suit‐Delclos due to stainless steel tube for high‐dose‐rate brachytherapy by Monte Carlo calculations. Brachytherapy. 2009; 8 (3): 318–23. 19217355 10.1016/j.brachy.2008.11.012

[4] Sina S , Faghihi R , Meigooni AS , Mehdizadeh S , Zehtabian M , Mosleh‐Shirazi MA . Simulation of the shielding effects of an applicator on the AAPM TG‐43 parameters of CS‐137 Selectron LDR brachytherapy sources. Iran J Radiat Res. 2009; 7 (3): 135–40.

[5] Fragoso M , Love PA , Verhaegen F , et al. The dose distribution of low dose rate Cs‐137 in intracavitary brachytherapy: comparison of Monte Carlo simulation, treatment planning calculation and polymer gel measurement. Phys Med Biol. 2004; 49 (24): 5459–74. 15724536 10.1088/0031-9155/49/24/005

[6] Grigsby PW , Williamson JF , Perez CA . Source configuration and dose rates for the Selectron afterloading equipment for gynecologic applicators. Int J Radiat Oncol Biol Phys. 1992; 24 (2): 321–27. 1526871 10.1016/0360-3016(92)90688-e

[7] Moslemi V , Esmaili‐Torshabi A , Mosleh‐Shirazi MA , et al. CT‐based brachytherapy treatment planning using Monte Carlo simulation aided by an interface software. Iran J of Med Phys. 2011; 8 (30): 41–53.

[8] van der Laarse R . A dedicated Selectron treatmeant planning system (SPS). Amsterdam, Netherlands: Antoni Van Leeuwenhoek Hospital, Netherland Cancer Institute; 1985.

[9] Sina S . Simulation and measurement of dosimetric parameters for 137Cs brachytherapy source, based on TG‐43 protocol by TLD and Monte Carlo [thesis]. Shiraz, Iran: Shiraz University; 2007.

[10] Siwek RA , O'Brien PF , Leung PMK . Shielding effects of Selectron applicator and pellets on isodose distributions. Radiother Oncol. 1991; 20 (2): 132–38. 2031088 10.1016/0167-8140(91)90147-9

[11] Pérez‐Calatayud J , Granero D , Ballester F , Lliso F . A Monte Carlo study of intersource effects in dome‐type applicators loaded with LDR Cs‐137 sources. Radiother Oncol. 2005; 77 (2): 216–19. 16226817 10.1016/j.radonc.2005.08.007

[12] Sina S , Faghihi R , Meigooni AS , Mehdizadeh S , Mosleh Shirazi MA , Zehtabian M . Impact of the vaginal applicator and dummy pellets on the dosimetry parameters of Cs‐137 brachytherapy source. JACMP. 2011; 12 (3): 183–93. 10.1120/jacmp.v12i3.3480 PMC5718639 21844861

[13] Pérez‐Calatayud J , Granero D , Ballester F . Phantom size in brachytherapy source dosimetric studies. Med Phys. 2004; 31 (7): 2075–81. 15305460 10.1118/1.1759826

[14] Markman J , Williamson JF , Dempsey JF , Low DA . On the validity of the superposition principle in dose calculations for intracavitary implants with shielded vaginal colpostats. Med Phys. 2001; 28 (2): 147–55. 11243337 10.1118/1.1339224

[15] Meisberger LL , Keller RJ , Shalek RJ . The effective attenuation in water of the gamma rays of gold 198, iridium 192, cesium 137, radium 226, and cobalt 60. Radiology. 1968; 90 (5): 953–57. 5643598 10.1148/90.5.953

[16] Briesmeister JF, ed. MCNP – a general Monte Carlo N‐particle transport code, Version 4C. Los Alamos, NM: Los Alamos National Laboratory; 2000.

[17] Pérez‐Calatayud J , Granero D , Ballester F , Puchades V , Casal E . Monte Carlo dosimetric characterization of the Cs‐137 selectron/LDR source: evaluation of applicator attenuation and superposition approximation effects. Med Phys. 2004; 31 (3): 493–99. 15070245 10.1118/1.1644640

[18] Halperin EC , Perez CA , Brady LW . Principles and practice of radiation oncology, 5th edition Baltimore, MD: Lippicott Williams and Wilkins; 2008.

